# Origin and Evolution of Genes in Eukaryotes: Mechanisms, Dynamics, and Functional Implications

**DOI:** 10.3390/genes16060702

**Published:** 2025-06-12

**Authors:** Salvatore Saccone, Desiree Brancato, Francesca Bruno, Elvira Coniglio, Valentina Sturiale, Concetta Federico

**Affiliations:** 1 Department of Biological, Geological and Environmental Sciences, University of Catania, 95124 Catania, Italy; desiree.brancato@phd.unict.it (D.B.); elvira.coniglio@phd.unict.it (E.C.); valentina.sturiale@phd.unict.it (V.S.); concetta.federico@unict.it (C.F.); 2 Department of Medicine and Surgery, Kore University of Enna, 94100 Enna, Italy; francesca.bruno@unikore.it

**Keywords:** de novo gene birth, gene duplication, orphan genes, neofunctionalization, subfunctionalization, nuclear architecture, compositional genome organization, evolutionary innovation, selection and drift, functional diversification

## Abstract

The origin and evolution of genes are central themes in evolutionary biology and genomics, shedding light on how molecular innovations shape biological complexity and adaptation. This review explores the principal mechanisms underlying gene emergence in eukaryotes, including gene duplication, de novo gene birth, horizontal gene transfer, viral gene domestication, and exon shuffling. We examine the population dynamics that govern the fixation of new genes, their functional integration, and the selective forces acting upon them—from purifying selection to adaptive innovation. Examples such as *NOTCH2NL* and *SRGAP2C*, which originated through recent segmental duplications followed by neofunctionalization, illustrate how duplicate-derived de novo genes can play a key role in human brain development. In addition, we highlight the emerging relevance of nuclear architecture in determining the evolutionary fate of new genes, offering a spatial dimension to gene innovation. We also discuss methodological approaches for detecting new genes and inferring selection, and finally, we highlight the emerging role of the human pangenome in revealing hidden gene diversity and its implications for evolutionary and biomedical research. Understanding gene innovation not only enhances our grasp of evolutionary processes but also informs clinical studies on disease susceptibility and human uniqueness.

## 1. Introduction

The evolution of eukaryotic genomes is characterized by a dynamic balance between conservation and innovation. At the heart of this process lies the continuous origin of new genes, which provide the raw material for functional diversification, environmental adaptation, and the increase in biological complexity. Traditionally, genes have been considered stable and well-defined entities; however, modern genomic and transcriptomic analyses have revealed a much more fluid and modular landscape, in which gene birth and loss are frequent events, even over relatively short evolutionary timescales.

Throughout evolution, the emergence of new genes has played a fundamental role in shaping the molecular *architecture* of organisms, contributing to the appearance of novel phenotypic traits and metabolic pathways. These events are particularly relevant in eukaryotes, whose genomes exhibit exon–intron gene structures and a large proportion of non-coding DNA, both of which facilitate molecular innovation. In addition, the high degree of regulatory plasticity, the presence of transposable elements, and the propensity for genomic and sub-genomic duplications make eukaryotic genomes particularly fertile grounds for the emergence of new genes. Recent studies also suggest that nuclear genome organization and chromatin topology contribute to this dynamic, influencing the likelihood that new genetic elements are transcribed, regulated, and functionally retained.

This review aims to systematically explore the main molecular mechanisms involved in the formation of new genes in eukaryotes, with a particular focus on gene duplication, protein domain reshuffling, de novo birth from non-coding regions, and the fixation of these novelties within populations. Emblematic examples of expanding gene families will also be discussed, along with the selective role of functional innovations in mammals and the human species.

## 2. Mechanisms of Gene Emergence and Divergence

The origin of new genes represents one of the main sources of biological and evolutionary innovation. In eukaryotes, various molecular mechanisms contribute to the formation of novel genes or to the structural rearrangement of existing ones, promoting functional diversification and species adaptation.

### 2.1. Gene Duplication and Functional Divergence

One of the main mechanisms for the emergence of new genes is the duplication of a pre-existing gene. This process generates two identical, non-allelic copies that can be located either adjacently or distantly within the genome [1]. While one copy retains the original function, the other is free to accumulate mutations that may alter its function without compromising organismal viability. Evolutionary outcomes of the duplicated gene include neofunctionalization (acquisition of a novel function), subfunctionalization (partitioning of the ancestral function), or pseudogenization (loss of function) [2].

**Figure 1:**
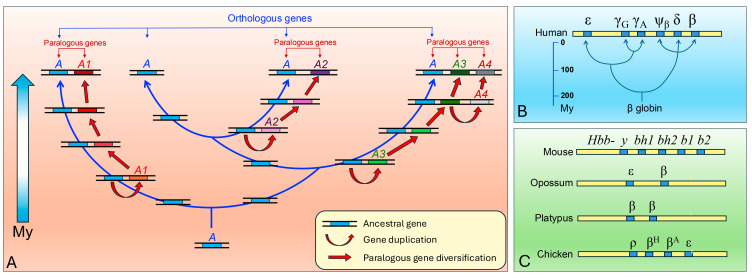
**The origin of new genes via sub-genomic duplication.** (**A**) A schematic representation of the duplication and divergence of a gene that originated in a common ancestor millions of years ago (My). All present-day species illustrated in the diagram retain at least one orthologous copy of the ancestral gene (labeled “*A*”), which performs the same function across lineages and maintains high sequence similarity. Along each evolutionary path, lineage-specific gene duplication events have produced additional paralogous copies. Initially, these paralogs retain the same function as the gene they were duplicated from, since they are identical in sequence at the moment of duplication; however, this function may differ from that of the original ancestral gene due to prior divergence, as exemplified by gene “*A4*” in the scheme, duplication from “*A3*”. Over time, lineage-specific mutation accumulation may lead to functional diversification of the paralogous copies through neofunctionalization, subfunctionalization, or pseudogenization. As a result, each extant species exhibits a unique set of paralogous genes, reflecting its specific history of gene duplication. Orthologous genes are defined as homologous genes in different species that derive from a common ancestral gene and retain the same function. Paralogous genes (*A1*, *A2*, *A3*, *A4* in the scheme) are gene copies within the same genome that originated through duplication events and may acquire divergent functions. (**B**) A schematic representation of the origin and chromosomal organization of the *β-globin* gene family in the human genome, arising from successive duplication events followed by sequence divergence. (**C**) *β-globin* gene clusters in mouse, opossum, platypus, and chicken, reconstructed according to the evolutionary framework illustrated in panel (**A**). The number and arrangement of paralogous copies vary across species, reflecting their distinct evolutionary histories. In each species, the cluster retains at least one orthologous gene (the *β-globin* gene) that preserves the ancestral function. Gene size and intergenic distances are not to scale. In the mouse, the full gene names are *Hbb-y*, *Hbb-bh1*, *Hbb-bh2*, *Hbb-b1*, *Hbb-b2*. Data adapted from ref. [8,9].

This dynamic has led to the formation of numerous gene families, in which paralogous genes share high sequence homology but perform partially distinct functions (Figure 1). A typical example is represented by globin genes, where duplication and divergence generated isoforms adapted to different physiological or environmental contexts [3,4]. The γ globin subunit of fetal haemoglobin, for instance, exhibits higher oxygen affinity than the adult β globin, optimizing gas exchange across the placenta [5]. In marine mammals, muscle myoglobin displays unique features that enhance oxygen storage during deep diving [6]. Similarly, adult hemoglobin’s in high-altitude-adapted species such as llamas exhibit the oxygen affinity properties of fetal forms [7]. These examples demonstrate how subtle variations in gene sequence can produce substantial biological effects and drive adaptation.

### 2.2. Exon Duplication

Beyond whole-gene duplication, structural mutations can lead to the formation of new genes through exon duplication or reshuffling, mechanisms facilitated by the modular architecture of eukaryotic genes [10]. Serial duplication of exons can result in the extension of a gene and the repetition of specific protein domains, potentially conferring novel structural or functional properties.

An emblematic case is the human gene *apo(a)*, involved in lipid transport, which originated from a duplication of the *plasminogen* gene [11]. During its evolution, the duplicated copy underwent expansion of the Kringle IV domain, encoded by a specific exon, producing variants with up to 50 repeats and marked inter-individual variability. These variants affect cardiovascular risk and illustrate how the expansion of protein domains can significantly influence physiology [12].

### 2.3. Retrotransposition

Retrotransposition is a process mediated by mobile elements, in which an mRNA transcript is reverse-transcribed into complementary DNA (cDNA) and reinserted into the genome. Genes arising through this mechanism, known as retrocopies, initially lack introns and native regulatory sequences, but may occasionally acquire functional promoters and become expressed [13]. Retrotransposition has generated numerous functional genes in mammals, with a higher frequency compared to other vertebrate lineages [14]. An illustrative example is *PGAM3*, a retrocopy of the glycolytic enzyme *PGAM1*, which has acquired testis-specific expression and may play a selective role in primate reproduction [15].

### 2.4. Gene Fusion and Fission

#### 2.4.1. Gene Fusion

Gene fusion occurs when parts of two or more previously separate genes combine into a single transcriptional unit, leading to the production of a chimeric protein with domains from different origins. This event can be driven by chromosomal rearrangements, such as translocations or deletions, or by transcriptional readthrough followed by selection [16]. The newly formed gene may acquire novel functions through the juxtaposition of functional domains not previously associated.

In vertebrates, an emblematic example is the *JAZF1-JJAZ1* gene fusion, frequently observed in endometrial stromal tumors, which generates a chimeric transcription factor with altered regulatory properties [17]. In evolutionary terms, the creation of new protein architectures through fusion events is a key driver of innovation. For instance, the *TRIM5-Cyclophilin A* (*TRIMCyp*) fusion gene in some New World monkeys confers resistance to HIV-1-like viruses [18], illustrating how gene fusion can lead to adaptive advantages.

#### 2.4.2. Gene Fission

Conversely, gene fission refers to the splitting of a single ancestral gene into two or more distinct genes. This can occur through deletion or insertion events that interrupt the original gene structure, followed by functional specialization of the derived units [19]. Although less common than fusion, fission can also contribute to genomic and functional diversification, as observed in some cases of metabolic gene evolution in bacteria and early eukaryotes.

### 2.5. Exon Shuffling

Exon shuffling is a molecular mechanism through which exons, particularly those encoding protein domains, are recombined between different genes. This process is facilitated by the modular nature of eukaryotic genes and is often mediated by recombination events involving intronic or intergenic regions. The outcome is the generation of new genes with novel combinations of functional domains, which may lead to innovative structural and biochemical properties [20].

**Figure 2:**
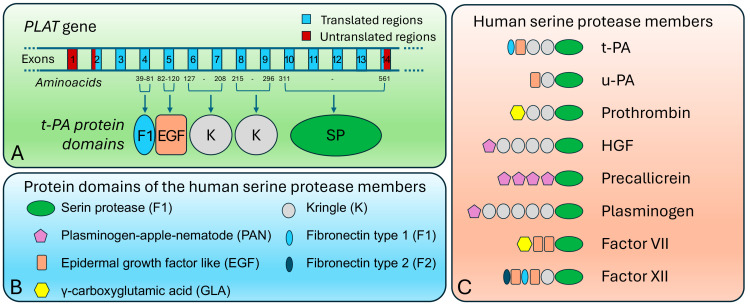
**Exon shuffling and human serine proteases.** (**A**) The structure of the human *PLAT* gene (NCBI RefSeq: NM_000930.5), which encodes the tissue-type plasminogen activator isoform 1 (*t-PA*; RefSeq: NP_000921.1). Exons are annotated according to the protein domains they encode. Individual domains are typically encoded by a single exon (e.g., F1 or EGF), by two adjacent exons (e.g., Kringle domains), or by multiple exons (e.g., the serine protease domain), depending on domain size. Protein domain annotations were retrieved from the UniProt database (https://www.uniprot.org/, accessed on 12 May 2025). Gene structure, exon size, and domain length are not drawn to scale. (**B**) A schematic overview of protein domains commonly found in human serine proteases. (**C**) Domain architectures of selected human serine proteases, illustrating distinct combinations of the domains depicted in panel (**B**).

Shuffling events are especially prevalent in metazoans, where complex exon–intron structures and the abundance of recombination-competent elements enhance the plasticity of gene architectures [21,22]. An iconic example is the formation of the tissue-type plasminogen activator (*tPA*) gene, which combines domains homologous to epidermal growth factor (EGF), fibronectin, and trypsin (Figure 2). This modular assembly underlies its dual function in fibrinolysis and cell signaling [23,24,25].

The evolutionary potential of exon shuffling lies in its ability to generate proteins with novel domain architectures, often with minimal deleterious impact, since recombination events typically occur within introns, preserving reading frames.

### 2.6. De Novo Gene Birth

De novo gene birth is the emergence of new protein-coding genes from previously non-coding DNA sequences. Unlike other mechanisms such as duplication or shuffling, which rely on pre-existing genes or gene fragments, de novo gene formation represents a radical innovation, as it involves the recruitment of entirely novel sequences into coding function [26].

This process typically requires the appearance of an open reading frame (ORF), the acquisition of transcriptional activity, and in some cases, the establishment of a translation initiation context. Although initially controversial, mounting genomic and transcriptomic evidence now supports the frequent and recurrent origin of de novo genes across diverse taxa, including humans [27,28].

Many de novo genes show tissue-specific expression and are often enriched in testes, suggesting roles in reproductive biology and potentially in speciation. For example, the human gene *FLJ33706* (now annotated as *C20orf62*, *NCBI Ref. Seq. NM_182584.4*) is considered a candidate de novo gene, arising from a previously non-coding intergenic region on chromosome 20 and exhibiting primate-specific expression patterns [29].

Although most de novo genes are short-lived and subject to rapid turnover, a subset may acquire essential functions and be retained by natural selection, highlighting their evolutionary significance.

### 2.7. Orphan Genes/Taxonomically Restricted Genes

Orphan genes are defined as genes lacking detectable homologues outside their reference taxon. These genes may originate from de novo events or from rapid divergence that obscures homology with other species. Orphan genes are often involved in taxon-specific biological functions, such as development, environmental adaptation, and reproduction [30].

In *Drosophila*, for instance, many orphan genes expressed during neural development or spermatogenesis appear to have evolved rapidly and may play fundamental roles in the evolution of genus-specific traits. These genes, while not conserved across species, contribute to the unique biology of the taxa in which they are found, potentially facilitating adaptations to specific ecological niches or reproductive strategies [31,32].

The study of orphan genes is important for understanding the genetic basis of species-specific characteristics and offers insights into how novel genetic innovations can arise and became fixed within populations.

### 2.8. Horizontal Gene Transfer

Horizontal gene transfer (HGT) refers to the non-vertical transmission of genetic material between organisms, bypassing traditional parent–offspring inheritance. While long recognized as a major evolutionary force in prokaryotes, its occurrence in eukaryotes—especially multicellular ones—has been more controversial and is typically limited to specific contexts. In unicellular eukaryotes, HGT has played a pivotal role in the acquisition of metabolic and stress-response genes, often from bacterial donors. In multicellular lineages, convincing cases of HGT are rarer but include gene acquisitions in bdelloid rotifers, fungi, and even vertebrate genomes [33,34].

A notable example in animals is the presence of microbial genes in the genome of the coffee borer beetle *Hypothenemus hampei*, which facilitates the digestion of caffeine and reflects a functional adaptation through HGT [35]. In humans, HGT-derived sequences are more difficult to detect due to strong vertical inheritance and genomic complexity, yet a few candidate events—particularly involving endogenous retroviral sequences—have been proposed to contribute regulatory elements and protein-coding exons [36,37,38]. Although not a widespread mechanism for new gene birth in complex eukaryotes, HGT nonetheless represents a potential source of innovation, particularly in the context of symbiosis interaction or genomic conflict.

### 2.9. Viral Gene Domestication as a Mechanism of Novel Gene Emergence

In addition to horizontal gene transfer, another important mechanism of gene emergence in mammals is the domestication of viral genes. Unlike canonical HGT events involving genes from bacteria or unicellular eukaryotes, viral gene domestication refers to the stable incorporation and functional co-option of viral sequences—especially those derived from endogenous retroviruses (ERVs)—into the host genome. These events have given rise to novel genes with essential roles in reproduction, development, immunity, and gene regulation [39].

A well-documented case is the domestication of retroviral envelope genes, known as syncytins, which have acquired key roles in placental development across mammalian lineages [40]. Other examples include gag-derived genes with regulatory or structural functions and reverse transcriptase domains repurposed in genomic regulation and transposition control. These domesticated elements are now recognized as a source of genetic novelty and evolutionary innovation, often exhibiting tight transcriptional regulation and tissue-specific functions [41].

This process of viral domestication represents a distinct route of gene emergence, shaped by ancient viral–host interactions and subject to strong selective constraints following functional integration.

### 2.10. Alternative Splicing

Alternative splicing is a major evolutionary innovation in eukaryotes, allowing a single gene to produce multiple transcripts and proteins. Its progressive diversification has significantly shaped gene function and organismal complexity throughout eukaryotic evolution. Early eukaryotes likely possessed few introns, but intron gain and loss events—particularly in metazoans and plants—have contributed to the structural and functional evolution of genes. The rise in alternative splicing in multicellular organisms enabled the production of multiple isoforms from a single gene, contributing significantly to proteomic complexity without increasing gene number. Notably, vertebrates exhibit extensive alternative splicing, with tissue- and development-specific patterns. This regulatory versatility has been implicated in the emergence of lineage-specific traits and in the evolution of brain complexity in primates [42].

A paradigmatic example of cell type-specific alternative splicing is provided by the DDX4 (VASA) gene, an evolutionarily conserved RNA helicase expressed in the germline across metazoans. In mammals, DDX4 undergoes alternative splicing generating isoforms with distinct expression patterns in spermatogonia, spermatocytes, and oocytes, modulating RNA metabolism in a stage-specific manner. Comparative analyses in other organisms such as Drosophila, Xenopus, and mammals reveal both conserved and divergent splicing events, suggesting that lineage-specific splicing patterns of germline genes contribute to the evolution of reproductive strategies and fertility mechanisms in eukaryotes [43,44].

A variety of molecular mechanisms underlie the birth of new genes in eukaryotes. These processes differ in origin, frequency, and functional consequences, and include gene duplication, de novo gene birth, horizontal gene transfer, and exon shuffling. The following Table 1 summarizes their main features, representative examples, and evolutionary outcomes.

These mechanisms have operated with varying prevalence across different eukaryotic lineages, contributing to lineage-specific genomic and phenotypic innovations. Table 2 illustrates how these mechanisms have shaped gene evolution in major eukaryotic groups, providing representative genes and references.

## 3. Population Fixation

Once originated, a new gene can follow different paths depending on the selective context and the evolutionary forces at play. Most new sequences do not reach fixation and are lost through genetic drift, negative selection, or simple transcriptional inefficiency. However, in some cases, a new gene provides even a minimal selective advantage, sufficient to promote its spread within the population [58]. The fixation of a new gene—that is, its stable retention and expansion within the species’ genetic pool—depends on several factors.

### 3.1. Mechanism of Fixation

#### 3.1.1. Positive Selection and Adaptation

Selective pressure is one of the primary drivers of gene fixation. Genes that provide even a minimal adaptive advantage can be rapidly selected, especially in small populations or those exposed to environmental stressors. This has been observed, for example, in genes involved in immune response, adaptation to new diets or environments, or brain development in the human lineage. In some cases, the fixation of a new gene may be accelerated by sexual selection, as observed for many testis-expressed genes that influence fertility or sperm competition. Positive selection promotes the rapid expansion of genes that improve the fitness of the organism [59,60].

#### 3.1.2. Contribution of Genetic Drift

In some circumstances, especially in small populations, a neutral or nearly neutral gene can fix even in the absence of selective advantages through genetic drift. This stochastic process can lead to the fixation of “passive” genes, which may later acquire important functions or become subject to selection. Genetic drift can be particularly significant in isolated or small populations, where the effect of random sampling is more pronounced. A gene that initially does not confer a selective advantage may become more common simply by chance, and if it later acquires a useful function, it may become subject to positive selection [61].

### 3.2. Biological Processes Facilitating Fixation

#### 3.2.1. Regulatory Context and Transcriptional Compatibility

For a new gene to be functional, it is not enough for it to be transcribed and translated: it is essential that it is expressed in the correct tissues, at the right times, and in a way that is consistent with pre-existing regulatory networks. Many new genes initially emerge as low-expression transcripts, often in the testes or other permissive tissues, where transcriptional activity is less stringent. This favorable environment allows the nascent gene to “test” its functionality with minimal risk to the organism [30,62].

Over time, if the new protein is not deleterious and acquires a beneficial function, regulatory mutations (in enhancers, promoters, or splice sites) may promote more stable or widespread expression. Mutations that enhance the gene’s expression in the correct tissues and at the right times are critical to its evolutionary success.

#### 3.2.2. Interaction with Pre-Existing Gene Networks

A new gene is more likely to fix if it can interact with existing molecular pathways, helping to modulate or enhance them. This functional integration can occur through the recognition of protein partners, incorporation into regulatory complexes, or interaction with RNA. In some cases, new genes act as modulators or regulators of pre-existing processes, even with initially performing redundant or accessory roles. Interaction with existing metabolic pathways or signaling systems increases the likelihood that the gene will be effectively integrated into the biological system, making its long-term retention more probable [30].

#### 3.2.3. Expansion Through Duplication Events

Even already-fixed genes can undergo further duplication, leading to the amplification or diversification of their function. A gene originally limited to a specific role or tissue expression may give rise to a gene family or paralogous set with diversified regulation and activity. These events contribute to increased functional complexity and adaptive potential. Gene duplication enables the testing of novel functions without compromising the ancestral one [63]. After duplication, gene copies may follow distinct evolutionary paths: neofunctionalization, with the acquisition of new roles [1]; subfunctionalization, where the original function is partitioned among duplicates [64]; or pseudogenization, leading to functional loss. Duplications may occur as tandem, segmental, or even whole-genome duplications, the latter playing a crucial role in early vertebrate evolution [65]. In plants, whole-genome duplications are particularly common and well tolerated, with both autopolyploidy (duplication within a single species) and allopolyploidy (duplication following hybridization between species) contributing significantly to plant diversification and ecological adaptation [66,67]. These mechanisms are evident in gene families such as the globins, which diversified to meet the oxygen transport demands of different developmental stages [3]. While often beneficial, gene duplication can also cause imbalances in gene dosage, contributing to disorders such as *MECP2* duplication syndrome. Comparative genomics continues to highlight how gene duplication drives evolutionary innovation, shaping both complexity and lineage-specific traits.

#### 3.2.4. Nuclear Architecture and Spatial Genome Organization Context

The spatial organization of the genome within the interphase nucleus has emerged as a key factor influencing not only gene expression patterns but also the evolutionary fate of newly arisen genes. Chromosomal regions located in gene-rich, transcriptionally active bands typically occupy more internal nuclear positions and are associated with euchromatic environments, favoring stable expression and functional integration [68]. Moreover, evolutionary studies indicate that such structural features—including chromatin topology and band-specific architecture—are conserved across vertebrates [69,70].

Recent research suggests that the chromatin and nuclear context in which a new gene emerges can strongly influence its evolutionary trajectory. Genes arising in GC-rich, euchromatic regions are more likely to become functional and fixed, whereas those in AT-rich, heterochromatic regions often remain silenced or are eventually lost [71,72,73]. Lamina-associated domains (LADs), typically located at the nuclear periphery, are enriched in repressive chromatin marks and correlate with low transcriptional activity [74,75]. Their association with the nuclear lamina may constrain the activation and retention of nascent gene sequences. During differentiation, however, some genes can reposition from LADs to more central euchromatic regions, acquiring transcriptional competence—a dynamic shift that may affect their long-term integration into functional networks.

This view is reinforced by research showing that gene-dense regions tend to associate with transcriptionally permissive nuclear sub-compartments such as transcription factories and euchromatic neighborhoods [76,77], whereas LADs and pericentromeric regions correlate with gene silencing and reduced activity [78,79]. These structural constraints may act as selective filters for gene innovation, favoring the emergence and persistence of new genes in spatially accessible and transcriptionally competent domains.

Altogether, these findings underscore the importance of considering nuclear architecture in evolutionary genomics. The interplay among three-dimensional genome organization, chromatin state, and gene emergence highlights a spatial dimension in the dynamics of gene birth and retention—an aspect increasingly relevant in light of single-cell and 3D genomics approaches.

## 4. Functional Innovation and Evolutionary Advantages

Once fixed in the population, a new gene can undergo various functional destinies depending on its origin, regulatory context, and protein interactions. The main trajectories for the functional evolution of new genes can be summarized as follows.

### 4.1. Subfunctionalization vs. Neofunctionalization

In the case of gene duplication, the two paralogs can specialize according to two main scenarios:

Subfunctionalization: Each copy maintains part of the original functions, for example, through spatial or temporal diversification of expression. This process is often driven by neutral mutations and may favor the preservation of both duplicated genes [64]. In this scenario, each copy performs a subset of the ancestral gene’s functions, potentially ensuring that neither copy is lost despite the redundancy in function.

Neofunctionalization: One copy acquires a novel function not present in the ancestral gene, for example, through mutations affecting active sites, functional domains, or regulatory regions [1,80]. This process is central to evolutionary innovation, as it enables the emergence of new functions that may confer a selective advantage to the organism.

### 4.2. Gene Expression and Post-Duplication Regulation

Regulatory divergence represents a primary pathway for innovation: even with identical coding sequences, modifications in promoters, enhancers, or epigenetic modulators can produce different expression patterns, leading to distinct functions in specific tissues or developmental stages [81,82]. The plasticity of new genes is particularly evident in rapidly evolving tissues, such as the brain and testes. This regulatory flexibility allows new genes to be adapted to specific biological contexts, enhancing their potential for functional diversification.

### 4.3. Epigenetic Regulation, Intrinsically Disordered Proteins, and Functional Plasticity

New genes are often subject to dynamic epigenetic regulation that plays a critical role in modulating their expression and evolutionary potential. Following duplication or origination, one gene copy can be temporarily silenced through DNA methylation **or** histone modifications, enabling an “incubation period” during which it accumulates beneficial mutations before being reactivated under specific signals or contexts [30,83]. This epigenetic flexibility ensures that gene activation remains tightly controlled and context-dependent, preventing deleterious effects while fostering adaptive innovation.

Moreover, many newly originated genes encode intrinsically disordered proteins (IDPs), characterized by the absence of a stable three-dimensional structure. These proteins are highly versatile, capable of interacting with numerous partners and performing moonlighting functions, i.e., multiple unrelated functions within the same cell [84,85]. Their structural flexibility complements epigenetic regulation by allowing the encoded proteins to adapt functionally in complex cellular networks, particularly in rapidly evolving tissues such as the brain and immune system.

Together, epigenetic modulation and the intrinsic disorder of many new proteins contribute to a high degree of functional plasticity. This dual mechanism facilitates the fine-tuning of gene expression and broadens the potential biological roles of new genes, promoting their successful integration and retention in the genome under varying environmental or developmental conditions.

## 5. Emblematic Examples in Vertebrates and Humans

The study of gene origin and diversification has led to the identification of numerous emblematic examples in vertebrates, particularly in humans, where the formation of new genes has played a pivotal role in the evolution of complex biological functions, the emergence of species-specific traits, and susceptibility to diseases. This section aims to highlight some of the most representative cases, with a particular focus on gene origin through duplications, de novo events, regulatory subfunctionalization, and adaptive innovations related to reproduction.

### 5.1. Expanded Gene Families

One of the clearest pieces of evidence for the origin and diversification of new genes is observed in expanded gene families, typically arising from duplication events followed by subfunctionalization or neofunctionalization. Olfactory receptors (ORs) represent the largest gene family in vertebrates, with humans possessing over 400 functional genes and an equal number of pseudogenes. Their expansion is related to the evolution of sensory strategies across diverse environments, such as olfactory specialization in rodents or the regression of olfactory function in higher primates, which has been offset by the development of trichromatic vision [86].

The *HOX* gene complex, which encodes transcription factors essential for anteroposterior embryonic development, has undergone multiple duplication events throughout vertebrate evolutionary history. The shift from a single cluster in protochordates to four clusters in vertebrates enabled greater modularity and complexity in body development, facilitating the evolution of specialized structures [87,88].

Another notable example is the *KRAB-ZNF* gene family, which includes over 350 genes in humans. The proteins encoded by these genes contain zinc finger domains coupled with KRAB domains, which mediate a repressive function. This family has expanded particularly in primates, as an evolutionary response to the proliferation of transposable elements, which are repressed through the recruitment of epigenetic cofactors such as TRIM28 [89].

### 5.2. Human-Specific Duplicated Genes with Novel Functions

Among the various mechanisms by which new genes originate, one of the most intriguing involves duplicated-derived gene birth, the emergence of protein-coding genes from ancestrally non-coding sequences. Unlike genes that arise through duplication and divergence, de novo genes represent truly novel genetic elements, often deriving from intergenic or intronic regions that gain transcriptional activity and, eventually, open reading frames. Although the functional validation of such genes remains challenging, growing evidence suggests that a subset of de novo genes is expressed in a tissue-specific manner—particularly in the brain—and is implicated in the evolution of the neocortex and advanced cognitive functions.

To illustrate the role of newly emerged genes in shaping species-specific traits, we present a selection of well-characterized, human-specific genes—*ARHGAP11B*, *NOTCH2NL*, and *SRGAP2C*—that exemplify distinct evolutionary origins, including partial and segmental duplications, as well as structural modifications that generate novel functions. These case studies have been chosen for their established contributions to the development and expansion of the human neocortex, highlighting how recent gene duplications and structural innovations can drive neurodevelopmental complexity and cognitive evolution.

*ARHGAP11B* is a human-specific gene that originated approximately 5 million years ago through a partial duplication of the ancestral gene *ARHGAP11A*. A subsequent point mutation introduced a novel splice donor site, resulting in a new coding exon and a truncated protein with distinct functional properties. It is predominantly expressed in basal progenitor cells within the developing human neocortex. Its expression promotes the proliferation of these progenitors, leading to an increased pool of neurons and contributing to the expansion of the neocortical surface area—a hallmark of the human brain. Ectopic expression of *ARHGAP11B* in the embryonic mouse neocortex has been shown to increase the number of basal progenitors and induce cortical folding (gyrification), a feature absent in the lissencephalic (smooth) mouse brain. These findings suggest that *ARHGAP11B* played a pivotal role in the evolutionary expansion and increased complexity of the human neocortex [54].

The *NOTCH2NL* gene family comprises human-specific paralogs—*NOTCH2NLA*, *NOTCH2NLB*, and *NOTCH2NLC*—derived from recent duplications of the ancestral *NOTCH2* gene. These duplications resulted in genes that retain the first few exons of NOTCH2 and acquire unique C-terminal sequences, leading to novel functional properties. These genes enhance Notch signaling, a pathway crucial for maintaining neural progenitor cells in a proliferative state. By promoting Notch activity, *NOTCH2NL* delays the differentiation of neural progenitors into neurons, thereby extending the period of cortical neurogenesis. This prolonged proliferative phase contributes to the increased neuronal output and the expansion of the human neocortex. Variations in the copy number of *NOTCH2NL* genes have been associated with neurodevelopmental disorders such as microcephaly and macrocephaly, underscoring their role in brain size regulation. The emergence of *NOTCH2NL* is believed to have been a key event in the evolution of the human brain, facilitating the development of its unique structural and functional features [55].

*SRGAP2C* is a human-specific paralog of the ancestral *SRGAP2A* gene, arising approximately 2.4 million years ago through an incomplete segmental duplication. This duplication resulted in a truncated version of *SRGAP2A* that retains the F-BAR domain but lacks the RhoGAP and SH3 domains. *SRGAP2C* dimerizes with *SRGAP2A*, acting as a dominant-negative inhibitor. This interaction inhibits *SRGAP2A*’s role in promoting dendritic spine maturation and limiting spine density. Consequently, *SRGAP2C* expression leads to increased spine density and prolonged periods of synaptic development (neoteny), features associated with enhanced synaptic plasticity and cognitive abilities in humans [90].

These examples illustrate how de novo genes can endow species with unique traits, highlighting their potential to influence complex biological processes such as brain development, neuronal differentiation, and cognitive function. In the human lineage, such genes may have contributed to the emergence of species-specific features, including the expanded neocortex and enhanced synaptic plasticity. Together, these findings underscore the remarkable capacity of new genes to acquire essential roles within a relatively short evolutionary timescale, reshaping our understanding of how genomic novelty drives phenotypic innovation.

### 5.3. Mammalian-Specific Duplicated Genes: Functional Divergence and Physiological Innovation

#### 5.3.1. Fetal Hemoglobin: Regulatory Subfunctionalization

The *HBG1* and *HBG2* genes, which encode the γ-globin chains of fetal hemoglobin (HbF, α₂γ₂), originated via a duplication event of an ancestral *β-globin* gene within the *β-globin* gene cluster located on human chromosome 11 (Figure 1) [8,9]. This duplication, which occurred early in the evolution of eutherian mammals, led to the formation of a multi-gene family that includes embryonic, fetal, and adult globin genes arranged sequentially and expressed in a developmentally regulated manner [91,92]. During fetal development, *HBG1* and *HBG2* are actively transcribed, producing γ-globin chains that, in combination with α-globin, form fetal hemoglobin. HbF has a higher affinity for oxygen than adult hemoglobin (HbA), allowing efficient oxygen transfer from maternal to fetal circulation—a critical adaptation for intrauterine life [93]. After birth, a switch in globin gene expression occurs: γ-globin expression is downregulated, and the adult *β-globin* gene (*HBB*) becomes the predominant transcript, resulting in the formation of HbA (α₂β₂).

This developmental switch is tightly regulated by epigenetic modifications, including DNA methylation and histone modifications, as well as by long-range interactions mediated by the locus control region (LCR) upstream of the *β-globin* cluster [94,95]. The silencing of *HBG* genes postnatally exemplifies a regulatory subfunctionalization process, wherein duplicated genes partition their expression domains—in this case, temporally—enabling fine-tuned physiological adaptation without requiring new coding functions. Moreover, this system represents a striking example of adaptive evolution following gene duplication, where changes in regulatory elements confer selective advantages, such as enhanced oxygen transport in the fetus—an essential trait in viviparous mammals [96]. The retention of both *HBG* genes, each with slightly different promoter sequences, also contributes to nuanced regulatory control and may influence HbF levels in adults, a feature of clinical relevance in disorders like β-thalassemia and sickle cell disease [97].

#### 5.3.2. Caseins: Reproductive Innovation in Mammals

Caseins (*CSN1S1*, *CSN2*, *CSN3*) are abundant milk proteins that facilitate the efficient transport of calcium, phosphorus, and amino acids to the neonate. Casein genes are specific to mammals and likely originated from duplications of ancestral genes involved in protein secretion.

This gene family has acquired new functions related to neonatal nutrition and is closely linked to the evolution of lactation, a trait exclusive to mammals. Caseins have also developed fine regulation in response to hormonal signals such as prolactin and glucocorticoids, contributing to a highly specialized system for offspring care [98]. This example illustrates how genetic evolution can contribute to the innovation of entire biological systems, with profound effects on physiology and reproductive behavior.

Caseins belong to the SCPP (secretory calcium-binding phosphoprotein) family, which also includes enamel proteins such as amelogenin (AMEL), ameloblastin (AMBN), and enamelin (ENAM). All of these genes originate from tandem duplications of a common ancestor and are located in clusters on human chromosome 4 [99].

Specifically, caseins appear to have evolved through two distinct evolutionary paths: the calcium-sensitive caseins (such as αS1-, αS2-, and β-casein) evolved from the *SCPPPQ1* gene, which is expressed in dental tissues and shares exonic structures with caseins, and the calcium-insensitive κ-casein evolved from the *FDCSP* gene, also expressed in dental tissues. Both of these genes ultimately derive from the ancestral *ODAM* (odontogenic ameloblast-associated) gene, expressed during enamel formation [100].

This origin suggests that caseins inherited the ability to bind calcium from their dental ancestors. Subsequently, these proteins were co-opted to form micelles in milk, a key innovation for neonatal nutrition in mammals. This represents an emblematic example of evolutionary co-option, where pre-existing genes are adapted to new biological functions.

## 6. From Origin to Function: Methods for Investigating the Emergence of New Genes

The study of gene origin requires the integration of genomic, transcriptomic, phylogenetic, and functional techniques. Thanks to the rapid development of high-resolution technologies and predictive power, it is now possible to analyze not only the emergence of new genes but also their expression, function, and evolutionary impact.

### 6.1. Comparative Genomics, Phylogeny, and Synteny

Comparative genomics enables the comparison of genomes across different species to identify orthologous genes, gene duplications, and new acquisitions. Gene phylogeny analysis helps reconstruct the evolutionary tree of genes, while synteny (the conservation of gene order between species) is crucial for recognizing de novo gene origins [101]. For instance, the human gene *ARHGAP11B***,** involved in cortical development, is absent in other primates and is located in a syntenic region with the ancestral *ARHGAP11A* gene, from which it originated through partial duplication followed by subsequent mutations [91]. This approach allows for the tracing of gene emergence over evolutionary time and provides insights into the mechanisms that drive innovation.

### 6.2. Transcriptomics and Ribosome Profiling (Ribo-Seq) for the Identification of New ORFs

RNA sequencing (RNA-seq) enables the identification of unannotated transcripts, potentially coding for new genes. However, mere transcription is not sufficient to establish gene functionality. Ribosome profiling (Ribo-seq) allows for the identification of which transcripts are actually translated, even in the case of small open reading frames (sORFs). Recent studies have shown that many regions previously annotated as non-coding actually produce functional micropeptides involved in cellular regulation [102]. This approach led to the definition of “proto-genes,” emerging transcripts that may evolve into fully functional genes. Together, these technologies are revolutionizing the study of gene function by allowing us to identify potential novel coding regions that were once considered non-coding.

### 6.3. Selection Analyses and Statistical Tests

To understand the evolutionary dynamics of genes, tests based on the ratio of non-synonymous to synonymous substitutions (dN/dS) are commonly employed. A dN/dS ratio greater than 1 suggests positive selection, whereas a ratio less than 1 indicates purifying selection. These analyses are typically performed using tools like PAML (Phylogenetic Analysis by Maximum Likelihood) (http://abacus.gene.ucl.ac.uk/software/paml.html, accessed on 18 May 2025) or HyPhy (Hypothesis testing using phylogenies) (https://www.hyphy.org/, accessed on 18 May 2025). Moreover, the integration of simulated-based models and Bayesian tests provides a robust estimate of selective pressures. Studies on genes like *SRGAP2C* have shown signs of positive selection in the human lineage, consistent with its functional acquisition in the control of neuronal migration [45]. This highlights how genetic innovations are shaped by natural selection to drive the emergence of new traits.

### 6.4. Experimental Technologies: CRISPR, In Vitro Models, and Organoids

Experimental approaches are crucial for determining the functional role of new genes. The CRISPR-Cas9 technology enables targeted gene knockout or the introduction of specific mutations to test their effects. In addition to CRISPR, classical gene knockout (KO) methods using embryonic stem (ES) cells have been extensively employed to generate animal models, particularly mice, allowing researchers to study gene function in vivo over developmental stages and adult physiology. These ES cell-based KO models have contributed to a vast accumulation of functional data on gene roles, including many involved in development and disease. In vitro models complement these genetic approaches. In particular, induced pluripotent stem cells (iPSCs) and brain organoids have allowed researchers to simulate human development in the laboratory. For instance, introducing *ARHGAP11B* into murine neuronal progenitors using gene editing or overexpression systems induced an expansion similar to that observed in the human cortex, suggesting a causal role in cognitive enhancement [54]. Brain organoids, derived from iPSCs, enable the exploration of gene functions in three-dimensional neural tissue models, bridging the gap between cell culture and whole-organism studies. This highlights the power of combining gene editing and cellular models to investigate the functional significance of new genes in developmental and evolutionary contexts.

Together, these experimental methods, ranging from traditional KO models using ES cells to cutting-edge CRISPR technology and advanced in vitro systems, enable comprehensive functional analyses of new genes allowing the comprehensive tracking of a new gene’s journey, from its appearance to its fixation in the population and the acquisition of a new biological function.

The following Table 3 provides an overview of the main technologies currently employed to study novel genes, highlighting their applications and representative examples.

## 7. Conclusions and Future Perspectives

The study of the origin and evolution of genes is not only a fascinating narrative about the ingenuity of nature but also a powerful lens through which we can understand the foundations of biological complexity and our very identity as a species. Analyzing the mechanisms that generate new genes—from duplication and de novo expression to the reorganization of genomic elements—offers critical insights into how new biological functions and innovative adaptations have developed over the course of evolution [27,108].

However, many questions remain unanswered:

• What is the actual frequency of de novo gene origin in different taxa, and what is their evolutionary stability?
• What traits emerge as a direct consequence of the appearance of new genes, and which ones are indirect effects on pre-existing gene networks?
• How can we distinguish between a true new functional gene and a “noisy” transcript lacking biological significance?

In the field of evolutionary biology, these questions touch the core of our understanding of speciation, adaptation, and morpho-functional innovation processes. In the biomedical field, identifying recently originated genes could offer new perspectives on complex diseases, human-specific traits, and even the development of targeted therapies. Some recent human genes, such as *NOTCH2NL* or *SRGAP2C***,** are strongly involved in cortical development, suggesting a direct link between genetic innovation and the evolution of higher cognitive abilities [45,109]. Others, like variants of the *apo(a)* gene, highlight the intersection between gene duplication and cardiovascular risk, serving as a striking example of the clinical potential impact of evolutionary research [110].

The integration of comparative genomics, epigenomics, structural biology, and systems biology will be essential for fully exploring the hidden potential in new genes and understanding their actual functional relevance. Next-generation sequencing technologies, genomic editing (such as CRISPR), and analysis in advanced model systems (organoids, humanized animals) are already revolutionizing this field. In particular, organoid-based platforms represent promising tools for dissecting the functional roles of novel genes in a tissue- and cell type-specific context, enabling experimental modeling of human-specific traits and pathologies. These approaches are supported by high-resolution reference datasets of human genetic variation [111], enabling the testing of functional hypotheses and biological causalities that were previously inaccessible.

An additional promising area for research is the study of nuclear architecture and chromatin spatial organization, which may influence both the expression potential and the evolutionary fate of newly emerged genes. Evidence suggests that genes located in gene-rich chromosomal bands or within chromatin territories localized in the more internal compartment of the cell nucleus are more likely to achieve stable expression and functional integration [68]. The conserved three-dimensional structure of chromosomal regions involved in evolutionary rearrangements underscores the critical relationship between genome topology and functional innovation, further highlighting how spatial positioning within the nucleus can shape the long-term retention and evolutionary potential of new genes [70].

A particularly promising direction for future research is the study of the human pangenome, which encompasses the complete genetic diversity within our species, beyond the single reference genome. Traditional genomic approaches have been largely based on a limited number of individuals, often from populations of European ancestry, thus failing to capture the full extent of global human diversity. The pangenome initiative aims to correct this bias by incorporating genomes from multiple individuals representing diverse ancestries, geographic origins, and evolutionary histories. Pangenome analysis is already revealing unexpected levels of structural variation, including insertions, deletions, inversions, and copy number variants, many of which are absent from the current reference genome. Importantly, it is also uncovering previously unannotated genes and novel isoforms, some of which may be population-specific or even individual-specific. To better understand the evolutionary dynamics of these new genetic elements, emerging single-cell multi-omics technologies—combining transcriptomics, epigenomics, and proteomics at cellular resolution—offer the opportunity to reconstruct their lineage-specific expression patterns, regulatory integration, and functional emergence across diverse cell types. These findings have far-reaching implications for both evolutionary biology and medicine. For example, population-specific genes may provide insights into local adaptation, while structural variants may contribute to disease susceptibility or resistance in ways that were previously invisible to reference-based analyses [112,113]. This global perspective allows for the rediscovery of new genes, understanding alternative expression patterns, and identifying potentially functional structural variants that were overlooked in classical genomic models.

Future research directions should include the following:

• Systematic functional characterization of novel genes in organ-specific or development-specific organoids.
• Integration of multi-omics data (transcriptome, epigenome, chromatin topology) at single-cell level to trace gene emergence across cell lineages.
• Investigation of how nuclear architecture affects expression potential of lineage-specific genes.
• Expansion of functional screens (e.g., CRISPR-based perturbation) targeting unannotated regions discovered through pangenome analysis.
• Evaluation of population-specific gene functions in health and disease using comparative organoid or xenograft models.

The pangenome thus represents a key tool for deepening our understanding of the origin and evolution of genes, enhancing the equity of biomedical research by including global human diversity, and improving our ability to interpret the genetic basis of health and disease.

The future of evolutionary genetics will increasingly unfold within an interdisciplinary landscape where genomics, evolutionary biology, computational modeling, developmental biology, and clinical sciences converge. It is precisely at the intersection between evolutionary history, molecular innovation, and clinical application that one of the greatest challenges (and promises) of modern biology lies: understanding not only where we come from but also where we can go.

## Figures and Tables

**Table 1:** Major mechanisms of gene birth: features, examples, and evolutionary roles.

Mechanism	Key Features	Representative Example	Functional Outcome	Refs.
Gene Duplication	Copy of existing gene; allows divergence	SRGAP2C	Neofunctionalization in brain development	[45]
De Novo Gene Birth	Origin from non-coding DNA	FLJ33706 (human)	Novel transcriptional regulator	[27]
Horizontal Gene Transfer	Transfer from other species	ACD in rotifers	Acquisition of stress resistance functions	[46]
Viral gene domestication	Incoroporation of viral sequences	Syncitin, ARC, PEG10	Novel gene emergence with essential roles	[37]
Exon Shuffling	Recombination of coding exons	Tissue plasminogen activator	New protein domains/functions	[47]

**Table 2:** Summary of gene evolution mechanisms across major eukaryotic lineages.

Eukaryotic Lineage	Predominant Gene Origin Mechanisms	Notes on Novelty and Expansion	Representative Genes	Refs.
Unicellular eukaryotes	Gene duplication, horizontal transfer	Limited novelty; compact genomes	*VSG* (Trypanosoma), *RBCS2* (Chlamydomonas)	[48,49]
Basal metazoans	Exon shuffling, duplication	Emergence of signaling and adhesion genes	*AIG1-like*, *GPCRs* (Amphimedon)	[50,51]
Invertebrates	Alternative splicing, duplication	Increased transcriptomic diversity	*DSCAM* (Drosophila), *FOG-2* (C. elegans)	[52,53]
Vertebrates	Gene duplication (WGD), de novo, viral gene domestication	Rapid expansion of lineage-specific genes	*ARHGAP11B*, *NOTCH2NL*	[37,54,55]
Plants	Polyploidy, de novo, duplication	High retention of duplicates, gene neofunctionalization	*FLC*, *LEC1* (Arabidopsis)	[56,57]

**Table 3:** Technologies for studying novel genes and illustrative examples.

Technology	Application	Example	Refs.
Comparative genomics and synteny	Identification of lineage-specific genes through genome comparison	*ARHGAP11B* evolution via duplication from *ARHGAP11A*	[54]
RNA-seq and Ribo-seq	Detection of novel transcripts and translated micropeptides	Discovery of functional sORFs and proto-genes	[103]
dN/dS analyses	Assessment of selective pressure on emerging genes	Positive selection on *SRGAP2*	[90]
CRISPR-Cas9 and ES cell KO models	Functional validation in animal and cell models	*ARHGAP11B* function in cortical development	[104]
Organoids (brain, iPSCs)	3D modeling of gene function in tissue-specific contexts	Human-specific neurodevelopmental traits	[105]
Human pangenome and SV discovery	Identification of population-specific new genes and structural variants	Unannotated genes and isoforms via pangenome	[106]
Single-cell multi-omics	Functional characterization of novel genes across cell types	Trajectory reconstruction of novel gene expression	[107]

## Data Availability

No new data were created or analyzed in this study. Data sharing is not applicable to this article.

## Abbreviations

The following abbreviations are used in this manuscript:

AMBN	Ameloblastin
AMEL	Amelogenin gene
*Apo(a)*	*Apolipoprotein A*
ARHGAP11A	Rho GTPase Activating Protein 11A
ARHGAP11B	Rho GTPase Activating Protein 11B
CSN1S1	Casein α S1
CSN2	Casein β
CSN3	Casein Kappa
ENAM	Enamelin
F-BAR	Fes/CIP4 homology-Bin/Amphiphysin/Rvs (F-BAR) domain proteins
FDCSP	Follicular Dendritic Cell Secreted Protein
*FLJ33706*	*Human-specific de novo protein-coding gene (alternative gene symbol C20orf203)*
HbA	Adult hemoglobin
HBB	Hemoglobin Subunit β
HbF	Fetal hemoglobin
HBG1	Hemoglobin Subunit γ-1
HBG2	Hemoglobin Subunit γ 2
HGT	Horizontal gene transfer
*HOX*	homeobox genes
IDPs	Intrinsically disordered proteins
iPSCs	Induced pluripotent stem cells
*JAZF- JJAZ1*	Zinc-Finger genes
KRAB-ZNF	Krüppel-associated box zinc finger
LADs	Lamina-associated domains
LCR	Locus Control Region
NOTCH2NL	Human-specific gene related to the Notch signaling pathway
NOTCH2NLA	Notch 2 N-terminal Like Protein A
NOTCH2NLB	Notch 2 N-terminal Like Protein B
NOTCH2NLC	Notch 2 N-terminal-Like Protein C
ODAM	Odontogenic ameloblast-associated gene
ORF	Open Reading Frame
Ors	Olfactory receptors
PAML	Phylogenetic Analysis by Maximum Likelihood
*PGAM3*	*Phosphoglycerate Mutase Family 3*
Ribo-seq	Ribosome profiling
RNA-seq	RNA sequencing
SCPP	Secretory calcium-binding phosphoproteins
SCPPPQ1	Secretory Calcium-Binding Phosphoprotein Proline-Glutamine Rich 1
SH3	Src Homology 3 domains
sORFs small	Open Reading Frames
SRGAP2A	SLIT-ROBO Rho GTPase Activating Protein 2
SRGAP2C	SLIT-ROBO Rho GTPase Activating Protein 2C
TRIMCyp	*TRIM5-Cyclophilin A*
